# Grey matter covariation and the role of emotion reappraisal in mental wellbeing and resilience after early life stress exposure

**DOI:** 10.1038/s41398-022-01849-6

**Published:** 2022-02-26

**Authors:** Haeme R. P. Park, Yann Quidé, Peter R. Schofield, Leanne M. Williams, Justine M. Gatt

**Affiliations:** 1 Neuroscience Research Australia, Sydney, NSW Australia; 2 School of Psychology, University of New South Wales, Sydney, NSW Australia; 3 School of Psychiatry, University of New South Wales, Sydney, NSW Australia; 4 School of Medical Science, University of New South Wales, Sydney, NSW Australia; 5 Psychiatry and Behavioral Sciences, Stanford School of Medicine, Stanford University, Stanford, CA USA

**Keywords:** Neuroscience, Psychology

## Abstract

Resilience is a process of adaptive recovery crucial in maintaining mental wellbeing after stress exposure. A psychological factor known to buffer stress and promote positive wellbeing outcomes is the ability to regulate emotions. However, the neural networks underlying resilience, and the possible mediating role of emotion regulation, remain largely unknown. Here, we examined the association between resilience and grey matter covariation (GMC) in healthy adults with and without early life stress (ELS) exposure, and whether emotion regulation mediated this brain-resilience association. Source-based morphometry was used to identify spatial patterns of common GMC in 242 healthy participants. Wellbeing was measured using the COMPAS-W Wellbeing Scale. Linear mixed models were run to establish associations between GMC and wellbeing scores. Moderated mediation models were used to examine a conditional mediating effect of emotion regulation on the brain-wellbeing relationship, moderated by ELS exposure. Distinct ELS-related morphometric patterns were found in association with resilience. In participants without ELS exposure, decreased GMC in the temporo-parietal regions was associated with wellbeing. In participants with ELS exposure, we observed increased patterns of covariation in regions related to the salience and executive control networks, and decreased GMC in temporo-parietal areas, which were associated with resilience. Cognitive reappraisal mediated the brain-wellbeing relationship in ELS-exposed participants only. Patterns of stronger GMC in regions associated with emotional and cognitive functioning in ELS-exposed participants with high levels of wellbeing may indicate possible neural signatures of resilience. This may be further heightened by utilising an adaptive form of emotion regulation.

## Introduction

Early life stress (ELS) is a risk factor for multiple adverse outcomes in adulthood, including increased risk for psychopathology, substance dependence, and suicide [1–3]. Alterations in brain structure have been associated with traumatic life experiences (including ELS) in the anterior cingulate cortex, amygdala, hippocampus, precuneus, and caudate nuclei [4–6]. Yet, many individuals are able to effectively compensate for such events and positively adjust to the potential neural changes elicited by ELS—defined as the resilience process [7]. Due to the positive implications of fostering resiliency in individuals, a growing number of neuroimaging studies have started exploring the neural correlates that may be related to increased resilience [3, 8–10]. However, the underlying structural bases that link both ELS exposure and mental wellbeing, and which may lead to a better understanding of the potential compensatory structural changes that are involved in resilience, remain unclear. Using a multivariate approach, this study aimed to determine the relationship between brain morphological networks and wellbeing/resilience as a function of ELS exposure, in a sample of healthy adults.

Resilience is a dynamic process encompassing both the attenuation of wellbeing disturbance and swift recovery after exposure [11]. It is now generally accepted that it is more than the mere absence of psychopathology [12, 13], and there are numerous factors that modulate or mediate an individual’s level of resilience, which may be measured as their level of wellbeing post-exposure (e.g. “how high is their level of wellbeing considering their previous ELS exposure?”) [14]. Although there is a large body of work reporting structural and functional changes after ELS exposure in otherwise ostensibly healthy adults [4, 15–18], the relationship between such neural differences and levels of wellbeing/resilience remains largely unknown. In a cohort of healthy adults, ELS-exposed individuals with high levels of wellbeing (therefore considered resilient) displayed reduced grey matter volume in the pons, suggesting there may be compensatory neural mechanisms involved in the resilience process that work to counter the negative effects of early life stressors [3]. Other studies have implicated the amygdala, insula, hippocampus, prefrontal cortex, and the anterior cingulate cortex as possible neural bases using voxel-based morphometry [19, 20]. However, this univariate method is unable to provide information regarding the relationships between voxels across the brain and covariation amongst multiple brain regions. As the potential effect of ELS and consequent development of resilience are likely to affect the whole brain rather than specific regions, a multivariate approach may be more sensitive in uncovering the relationship between wellbeing and larger brain networks of covarying voxels.

In addition to neural changes, psychological factors buffer stress and foster adaptive functioning, especially in relation to the way individuals respond to stressful situations. One particularly relevant construct is emotion regulation, where the type of strategy employed may have an effect on the individual’s level of resilience [21]. In more detail, an antecedent-focused strategy, such as cognitive reappraisal, acts to cognitively change our response to a stressful or emotional event, prior to the full activation of emotion response tendencies. On the other hand, a response-focused strategy, such as expressive suppression, works to modify the emotional response after it has already been triggered [22]. Previous studies have shown a positive association between reappraisal and mental health [23], while suppression was found to lead to maladaptive outcomes, such as depression and anxiety [24, 25]. Compared to trauma-exposed women with psychopathology, those without psychopathology showed stronger activation in the prefrontal cortex after being instructed to enhance their emotional reaction to a negative image [26] indicating a better ability to cope with negative stimuli via better emotional reappraisal, which may be integral to building resilience. However, the direct link between emotion regulation and the brain structure-wellbeing relationship is not yet known.

The goal of the current study was to examine the associations between structural networks and wellbeing/resilience as a function of ELS exposure in healthy adults. The first aim was to examine the interactions between brain networks, levels of wellbeing, and ELS exposure using source-based morphometry, which is a multivariate method that is able to identify naturally grouped patterns of structural networks [27]. We hypothesised that there would be structural changes across networks related to wellbeing in adulthood, driven by ELS exposure. However, due to a lack of studies focusing on structural networks related to levels of resilience in healthy adults, we made no predictions regarding the specific patterns that may be involved, although it is likely that regions such as the anterior cingulate cortex, hippocampus, and temporal regions will be affected. Our second aim was to examine the mediating effect of emotion regulation in this process given its implicated role in mental health and resilience. Here, we predicted that an increase in the use of the emotion regulation strategies would mediate the relationship between brain structural networks (especially in regions involved with emotion regulation, such as the prefrontal cortex [28]) and wellbeing levels, especially in participants exposed to ELS compared to those non-exposed. We also further hypothesised that the direction of this effect would differ depending on the type of emotion regulation strategy used (e.g. cognitive reappraisal versus expressive suppression) due to their opposing roles in mental health and wellbeing.

## Methods and materials

### Participants

Participants from the larger TWIN-E cohort study [29] that completed the MRI component were included in this current study. The initial sample consisted of 250 healthy same-sex monozygotic and dizygotic twins, without past/current psychiatric illness or traumatic brain injury based on self-report. Eight participants were excluded from the current analyses due to missing data, being an incomplete twin pair, or failing quality control after MR image preprocessing. The final sample for this study consisted of 242 participants (see Table 1). All participants gave their written informed consent to participate. The study received approval from the Human Research Ethics Committee of the University of Sydney (03–2009/11430), in accordance with the Helsinki Declaration of 1975, and revised in 2008.

**Table 1:** Demographic characteristics for the final sample.

Measure	No ELS (*n* = 61)	ELS (*n* = 181)	Total (*n* = 242)
Zygosity (MZ/DZ)	38/23	125/56	163/79
Age (years ± SD)	38.8 (±13.3)	39.7 (±12.7)	39.5 (±12.9)
Sex (M/F)	27/34	61/120	88/154
COMPAS-W total (range: 26–130; ±SD)	100 (±9.50)	99.1 (±11.3)	99.3 (±10.9)
DASS-42 total (range: 0–126; ±SD)	8.77 (±7.34)	11.8 (±12.2)	11.1 (±11.2)
ERQ reappraisal (range: 0–6; ±SD)	5.01 (±1.07)	5.06 (±1.06)	5.04 (±1.06)
ERQ suppression (range: 0–4; ±SD)	3.51 (±1.02)	3.56 (±1.27)	3.55 (±1.21)

Total number of the sample reflects the final sample of participants included in all analyses (SBM, linear mixed models, moderated mediation model).

*ELS* early life stress exposure identified from the Early Life Stress Questionnaire, *MZ* monozygotic twin, *DZ* dizygotic twin, *COMPAS-W* total composite measure of wellbeing, *DASS-42* total measure of negative mood symptoms of depression, anxiety, and stress, *ERQ* measure of emotion regulation that includes reappraisal and suppression subscales.

### Psychometric measures

Four self-report psychometric scales were used to measure wellbeing (the COMPAS-W scale of wellbeing [30]), negative mood symptoms (DASS-42 [31]), early life stress exposure (Early Life Stress Questionnaire [32]), and emotion regulation strategies (Emotion Regulation Questionnaire [33]). In brief, the COMPAS-W is a composite scale that measures both subjective and psychological subcomponents of wellbeing, while the DASS-42 consists of items that measure depression, stress, and anxiety symptoms (collectively labelled as negative mood symptoms here). To ensure normality, z-scores for the COMPAS-W and log-transformed scores for the DASS-42 were used in the current study to index the participant’s levels of wellbeing and negative mood symptoms, respectively. The COMPAS-W score was taken as a score for levels of resilience in the participants with previous ELS exposure (i.e., adaptive recovery after adversity).

Exposure to early life stress was measured using the Early Life Stress Questionnaire, which uses a categorical ‘Yes’ or ‘No’ response to 19 early life stress events that may have occurred prior to the age of 16 years, such as physical/sexual/emotional abuse, neglect and poverty, health-related traumas, bullying, and family/parent-related conflict and separation (see Fig. S1 in the Supplementary Materials for the distribution of the number of events in our sample). Here, we dichotomised the current sample into either having had exposure to early life stress (‘Yes’ to any of the items) or no exposure (‘No’ to all of the items; but see Supplementary Materials S1 for a 0 vs 1–2 vs 3+ events comparison).

Lastly, emotion regulation strategies were measured using the Emotion Regulation Questionnaire, consisting of two subscales: cognitive reappraisal, which is an antecedent-focused strategy that has been linked with adaptive management of affect [33], and expressive suppression, which is a response-focused strategy that has been linked with poor social outcomes [34]. The average scores from each subscale were used in the current study.

### Image acquisition and preprocessing

Magnetic resonance images were acquired with a 3 T GE Signa HDx scanner (GE Healthcare, Milwaukee, WI, USA) equipped with an eight-channel head coil based at the Westmead Hospital Medical Imaging Service (Sydney, NSW, Australia). Three-dimensional (3D) T1-weighted volumes were acquired using a spoiled gradient echo (SPGR) sequence (TR = 8.3 ms; TE = 3.2 ms; flip angle = 11 degrees; inversion time = 500 ms; FOV = 256 mm; 180 sagittal slices; matrix size = 256 × 256; voxel size = 1 × 1 × 1 mm; NEX = 1; ASSET = 1.5; scanning time = 7.12 min).

The images were preprocessed using the Computational Anatomy Toolbox (CAT12.7, Structural Brain Mapping group, Jena University Hospital, Jena, Germany; http://dbm.neuro.uni-jena.de/cat12/) for SPM12 (v7487, Wellcome Trust Centre for Neuroimaging, London, UK; http://www.fil.ion.ucl.ac.uk/spm) running in MATLAB R2018b (MathWorks, Natick, MA). After correcting for bias field inhomogeneities using the CAT12 default setting (medium), the images were segmented into grey matter (GM), white matter, and cerebrospinal fluid using a classic unified segmentation algorithm. These were then modulated and normalised to Montreal Neurological Institute (MNI) space, then resampled to a voxel size of 1.5 × 1.5 × 1.5 mm. Quality control of processed images was performed by checking for image homogeneity, using the Mahalanobis distance between the mean correlations of the scans and the weighted overall image. From this, four participants were identified as outliers, from which one participant (and his twin) were excluded from further analyses after a careful visual inspection of the scans. Finally, the images were smoothed using an isotropic Gaussian filter of 8 × 8 × 8 mm at full-width half-maximum. Total intracranial volumes of each participant were also calculated in order to correct for brain size differences.

### Source-based morphometry analysis

Source-based morphometry (SBM) analysis was performed via the Group ICA for fMRI Toolbox (GIFT; [27] http://trendscenter.org/software/gift/). In order to derive the number of independent components, Infomax algorithm was used to identify maximally independent sources without any a-priori information regarding the nature of the signals. Each GM image was transformed into a one-dimensional vector, which was then placed into a subject-by-voxel matrix. This matrix was then decomposed into a mixing matrix that quantifies the contribution of each subject to each component (subjects-by-components that produce loading coefficients), and a source matrix that represents the extent to which each voxel contributes to a spatially independent component, based on morphometric covariance within the cohort (components-by-voxels that produce spatial components). The ‘minimum description length’ was used to estimate the number of components, and the ICASSO bootstrapping algorithm was used to increase the stability of the estimated components (20 iterations). Each spatial component was reshaped back into a three-dimensional image, normalised to unit standard deviations, and thresholded at |z | > 2.5 for visualisation. Talairach labelling utility within the GIFT toolbox was used to label all resulting clusters, with a minimum cluster size of 1 cm^3^.

### Linear mixed model and moderation analyses

To examine the initial associations between ELS exposure, wellbeing, and the loading coefficients derived from SBM, linear mixed models were performed using *lme4* package in *R*, with wellbeing being the outcome variable, while loading coefficients from each component, ELS exposure status, and their interaction were entered as fixed effects predictors. Age, sex, zygosity, DASS-42 total scores, and total intracranial volume (TIV) were included as covariates, while family ID was included as a random-effects predictor to control for relatedness (due to being a twin). Only the components found to be significantly (*p* < 0.05) associated with ELS-by-wellbeing interactions in this step were taken to moderation analyses to formally test the simple slope effects of ELS exposure, using *lavaan* in *R*. In separate models, the loading coefficients from each component were used as the predictors, wellbeing as the outcome, and ELS exposure as the moderator. Covariates included age, sex, zygosity, TIV, and the family ID.

### Moderated mediation analysis

Lastly, the mediating effect of emotion regulation strategies on the relationship between the SBM components and wellbeing was investigated, while taking into account the moderating effect of ELS exposure. After checking for associations between emotion regulation scores (mediator) and loading coefficients (predictor), *lavaan* in *R* was used to fit our model with 10,000 bootstrapped confidence intervals, with wellbeing scores as the outcome variable. The emotion regulation subscale was taken as the mediator between the loading coefficients and wellbeing, while ELS exposure was entered as the moderator on both the indirect (a) and direct (c’) paths (see Fig. 3A). This was to examine whether the potential moderated mediating effect via the indirect path would remain significant while controlling for the influence of a possible moderating effect of ELS exposure on the direct path.

## Results

### Demographics

Demographic characteristics for the final sample (*N* = 242) are summarised in Table 1. There were no significant differences between participants exposed or non-exposed to ELS for wellbeing, age, sex, zygosity, and ERQ reappraisal and suppression scores. However, those with ELS exposure scored significantly higher for the total DASS-42 total score (negative mood symptoms), compared to those without ELS exposure (*t*_*173.09*_ = −2.33, *p* = 0.021, *d* = 0.303).

### Source-based morphometry and linear mixed modelling results

Sixteen components were identified through ICA, with each component displaying a spatially distinct pattern of grey matter volume covariation across our sample (see Table S1 and Fig. S2 in the Supplementary Materials for a full description and depiction of the components). The linear mixed models identified that wellbeing was significantly associated with the interactive effects of ELS and loading coefficients from five independent components (IC2, IC5, IC10, IC11, and IC13; see Table 2).

**Table 2:** Linear mixed model results of the significant associations between wellbeing (COMPAS-W), independent components IC2, IC5, IC10, IC11, and IC13, and early-life stress exposure.

Effect	Estimate (β)	SE	t	*p*-value	CI
Wellbeing with IC2					
Intercept	1.44	0.909	1.58	0.115	[−0.313, 3.18]
Age	−0.015	0.006	−2.64	**0.009**	[−0.026, −0.004]
Sex	−0.011	0.150	−0.074	0.941	[−0.299, 0.277]
Zygosity	0.202	0.147	1.37	0.174	[−0.080, 0.484]
TIV	<−0.001	0.001	−0.043	0.966	[−0.001, 0.001]
IC2	−0.495	0.254	−1.95	0.052	[−0.988, −0.008]
ELS exposure	−0.005	0.140	−0.039	0.969	[−0.274, 0.266]
DASS-42	−1.02	0.143	−7.25	**<0.001**	[−1.30, −0.753]
ELS × IC2	0.284	0.141	2.01	**0.046**	[0.013, 0.560]
Wellbeing with IC5					
Intercept	1.58	0.950	1.66	0.099	[−0.259, 3.40]
Age	−0.013	0.006	−2.36	**0.020**	[−0.024, −0.003]
Sex	0.014	0.149	0.091	0.928	[−0.273, 0.300]
Zygosity	0.189	0.146	1.30	0.197	[−0.090, 0.470]
TIV	−0.001	0.001	−0.302	0.763	[−0.001, 0.001]
IC5	−0.663	0.266	−2.49	**0.013**	[−1.18, −0.153]
ELS exposure	0.001	0.139	0.006	0.996	[−0.266, 0.273]
DASS-42	−1.03	0.140	−7.32	**<0.001**	[−1.30, −0.757]
ELS × IC5	0.396	0.146	2.72	**0.007**	[0.117, 0.682]
Wellbeing with IC10					
Intercept	2.88	0.893	3.23	**0.001**	[1.16, 4.60]
Age	−0.010	0.006	−1.86	0.066	[−0.021, 0.001]
Sex	−0.009	0.144	−0.065	0.949	[−0.285, 0.266]
Zygosity	0.204	0.140	1.46	0.146	[−0.064, 0.473]
TIV	−0.001	0.001	−1.94	0.054	[−0.002, 0.001]
IC10	−0.482	0.261	−1.85	0.066	[−1.01, 0.019]
ELS exposure	−0.014	0.137	−0.101	0.920	[−0.276, 0.253]
DASS-42	−1.05	0.138	−7.60	**<0.001**	[−1.31, −0.782]
ELS × IC10	0.416	0.141	2.95	**0.003**	[0.146, 0.698]
Wellbeing with IC11					
Intercept	1.38	0.821	1.69	0.094	[−0.211, 2.96]
Age	−0.015	0.006	−2.65	**0.009**	[−0.026, −0.004]
Sex	<0.001	0.153	0.001	0.999	[−0.293, 0.293]
Zygosity	0.182	0.148	1.22	0.223	[−0.103, 0.467]
TIV	<0.001	0.001	0.145	0.885	[−0.001, 0.001]
IC11	−0.456	0.231	−1.98	**0.049**	[−0.899, −0.008]
ELS exposure	−0.052	0.144	−0.358	0.721	[−0.328, 0.230]
DASS-42	−1.01	0.141	−7.15	**<0.001**	[−1.28, −0.738]
ELS × IC11	0.283	0.132	2.15	**0.033**	[−0.028, 0.537]
Wellbeing with IC13					
Intercept	1.95	0.858	2.27	**0.024**	[0.298, 3.60]
Age	−0.012	0.006	−2.26	**0.026**	[−0.023, −0.002]
Sex	−0.003	0.147	−0.017	0.986	[−0.285, 0.280]
Zygosity	0.239	0.145	1.65	0.101	[−0.038, 0.518]
TIV	−0.001	0.001	−0.900	0.369	[−0.001, 0.001]
IC13	−0.432	0.243	−1.78	0.077	[−0.905, 0.035]
ELS exposure	−0.015	0.138	−0.105	0.916	[−0.280, 0.254]
DASS-42	−0.962	0.141	−6.84	**<0.001**	[−1.23, −0.691]
ELS × IC13	0.336	0.134	2.50	**0.013**	[0.078, 0.599]

*p*-values were derived using Satterthwaite approximations for degrees of freedom, which produce acceptable Type 1 error rates (Luke, 2017). Bold font denotes significant associations.

*TIV* total intracranial volume, *ELS* early life stress, *COMPAS-W* composite measure of wellbeing, *DASS-42* negative mood symptoms, *ELS × IC* interaction between early life stress exposure and the independent component, *SE* standard error, *CI* 95% confidence interval.

**Fig. 1:**
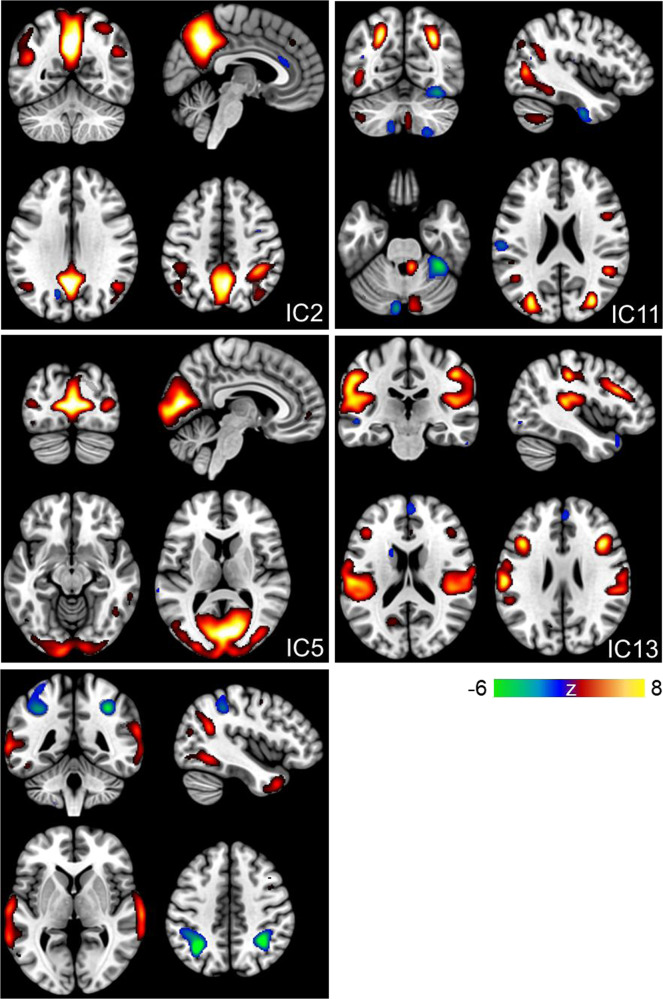
Spatial maps of the five independent components associated with wellbeing and showing a significant interaction with ELS exposure. Warm colours represent positive voxels for the component, while cool colours indicate negative voxels. The components were thresholded at |z | > 2.5; the colour bar indicates the z-value.

SBM results for these components revealed a pattern of positive and negative voxels. More specifically, for IC2, regions consisting of positive voxels (see Fig. 1 in warm colours) were clustered bilaterally around the superior temporal gyrus, inferior and superior parietal lobules, postcentral gyrus, precuneus, and the cingulate gyrus. There were no regions with negative voxels that survived the minimum cluster threshold. For IC5, positive voxels included the bilateral middle temporal and occipital gyri, left inferior occipital gyrus, precuneus, and posterior cingulate. Again, no regions survived the threshold for negative voxels. For IC10, positive voxels included bilateral inferior, middle, and superior temporal gyri, fusiform gyrus, and the precuneus, while negative voxels (see Fig. 1 in cool colours) were located in the bilateral inferior parietal lobule and parietal sub-gyral regions. For IC11, the network of regions consisting of positive voxels included the bilateral middle temporal gyri and precuneus, right inferior and left superior parietal lobules, right inferior and middle frontal gyri, postcentral gyrus, and the left middle occipital gyrus, while regions with negative voxels included the left inferior temporal gyrus, postcentral gyrus, and the right fusiform gyrus. Lastly, for IC13, there was a pattern of positive voxels in the bilateral insula, middle and superior temporal gyrus, inferior parietal lobule, precentral and postcentral gyri, and the middle frontal gyrus. Negative voxels of this IC were located in the left middle temporal and right medial frontal gyri.

### Moderation analyses

**Fig. 2:**
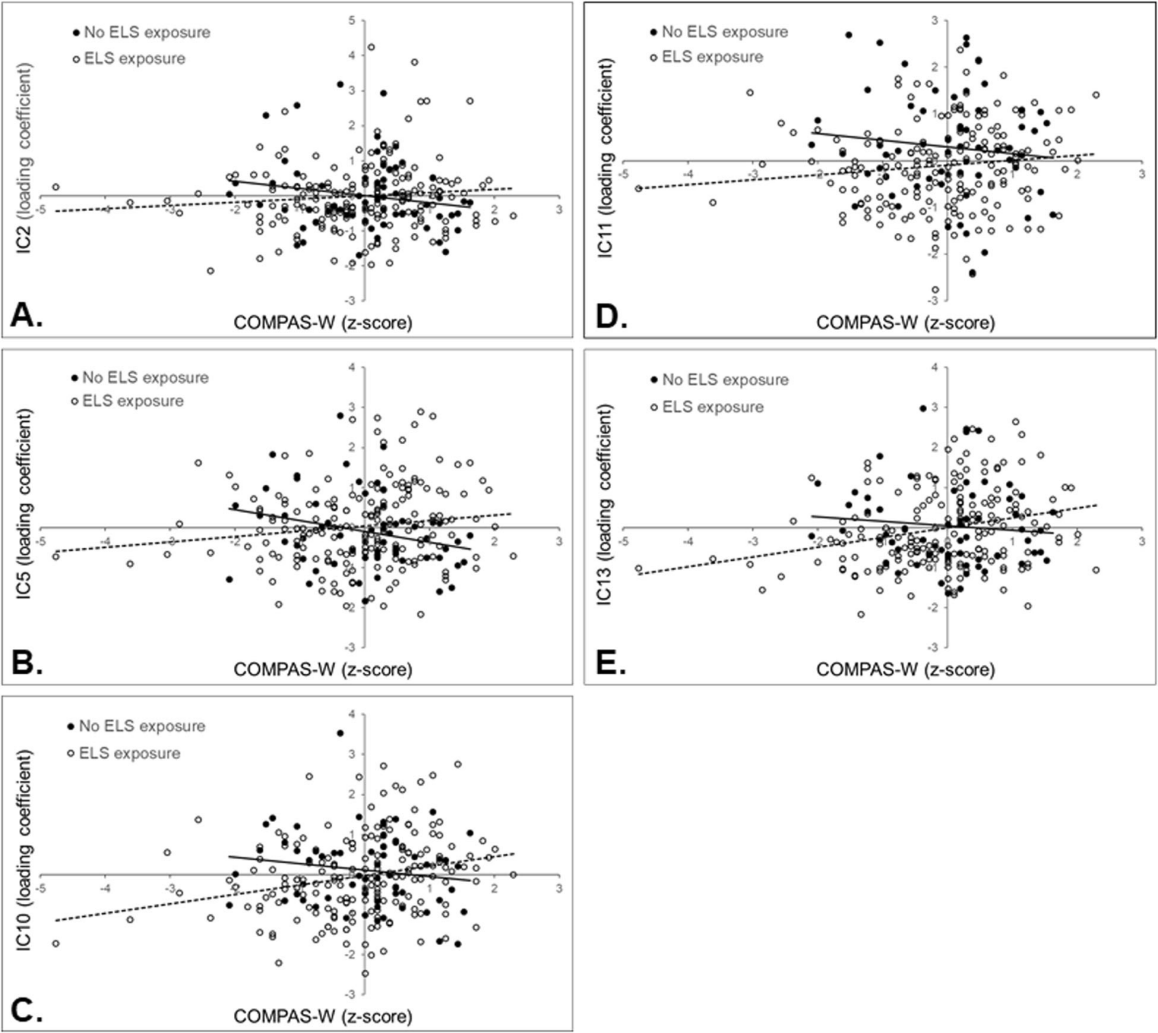
Graphs of the associations between source-based morphometry independent components (IC) and wellbeing. These associations are between IC2 (**A**), IC5 (**B**), IC10 (**C**), IC11 (**D**), and IC13 (**E**) and wellbeing (indexed by COMPAS-W *z*-scores) that showed an interaction with early-life stress (ELS) exposure. Participants without exposure showed a significant negative association between the GMC in each component and wellbeing (black dots; solid regression line) for IC2 and IC5, while those with previous exposure displayed a significant positive association between the GMC in each component and wellbeing (white dots; dotted regression line) for IC10 and IC13. The simple slopes for IC11 were not significant.

For IC2 and IC5, participants not exposed to ELS showed a significant negative association between wellbeing and the loading coefficients for these components (IC2: *b* = −2.36; SE = 0.884; *p* = 0.008; 95% CI [−4.09, −0.625]; IC5: *b* = −3.05; SE = 1.04; *p* = 0.003; 95% CI [−5.09, −1.01]), indicating decreasing GMC in the positive voxels (see Fig. 2A and 2B). For participants exposed to ELS, the relationship between GMC and wellbeing was not statistically significant (IC2: *b* = 1.09; SE = 0.950; *p* = 0.251; 95% CI [−0.773, 2.95]; IC5: *b* = 1.52; SE = 0.855; *p* = 0.074; 95% CI [−0.149, 3.20]). On the other hand, the results for IC10 and IC13 revealed a significant positive association between wellbeing and loading coefficients of these ICs for participants exposed to ELS, indicating that as their wellbeing levels increased, the GMC for these components also increased in regions consisting of positive voxels, and decreased in regions consisting of negative voxels (IC10: *b* = 3.92; SE = 0.885; *p* < 0.001; 95% CI [2.18, 5.65]; IC13: *b* = 2.78; SE = 0.832; *p* < 0.001; 95% CI [1.15, 4.41]; see Fig. 2C and 2E). The slopes were not significant for participants not exposed to ELS for these two components (IC10: *b* = −1.16; SE = 1.13; *p* = 0.302; 95% CI [−3.37, 1.04]; IC13: *b* = −1.09; SE = 0.846; *p* = 0.199; 95% CI [−2.75, 0.571]). For IC11, despite a significant interaction between the loading coefficients for this IC and ELS exposure on wellbeing, the simple slopes were not significant for either of the participants (non-exposed *b* = −1.47; SE = 0.907; *p* = 0.105; 95% CI [−3.25, 0.307]; exposed *b* = 1.37; SE = 0.819; *p* = 0.096; 95% CI [−0.240, 2.97]; Fig. 2D).

### Moderated mediation analyses

**Fig. 3:**
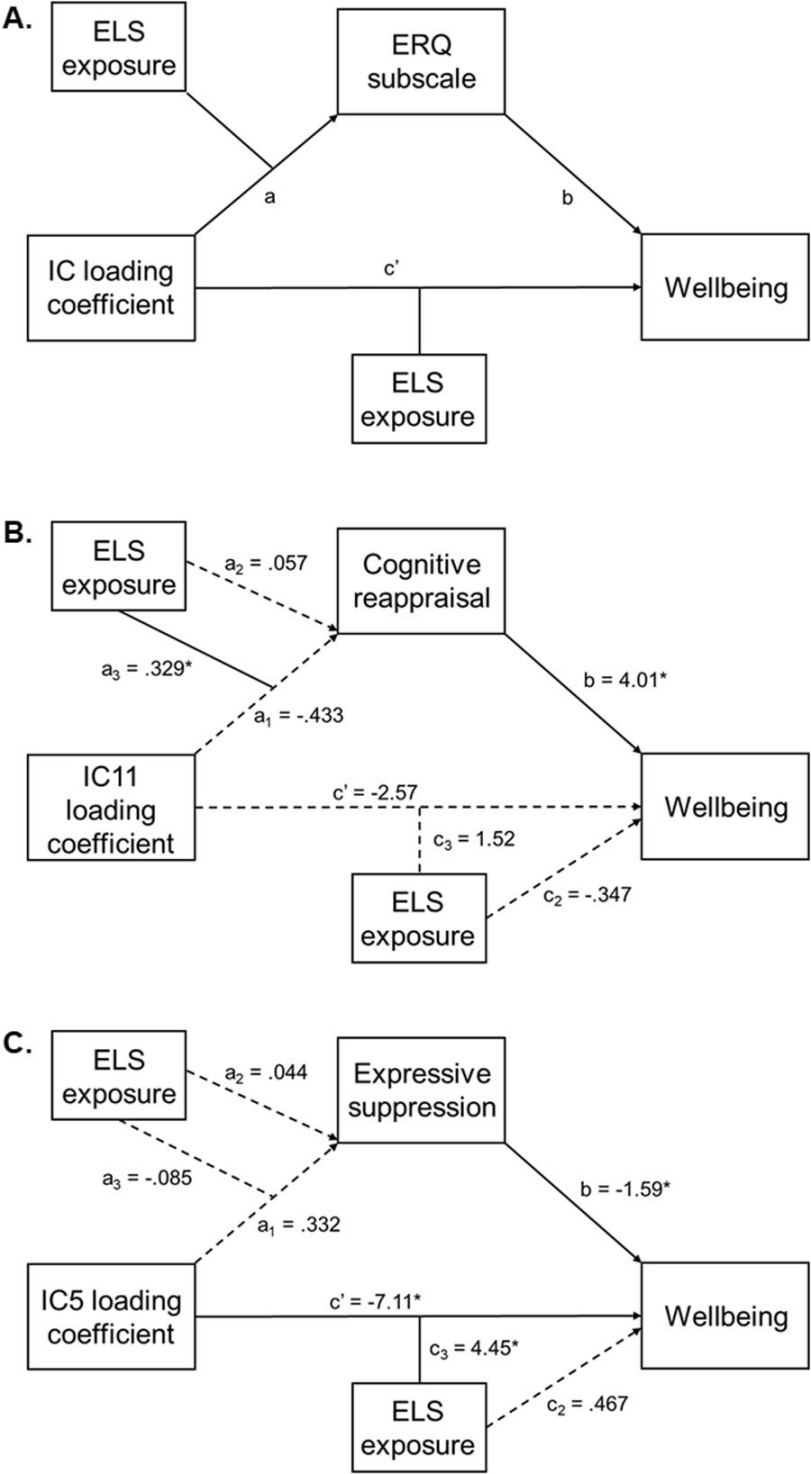
The conceptual and tested moderated mediation models. **A** The conceptual moderated mediation model shows the independent component (IC) loading coefficients as the predictor, the subscale of the Emotion Regulation Questionnaire (ERQ) as the mediator, and wellbeing (indexed by COMPAS-W scores) as the outcome. Early life stress (ELS) exposure was included as a common moderator for the associations between the SBM component and ERQ subscale scores (a path), and between the component and wellbeing scores (c’ path). **B** The moderated mediation model testing for the conditional indirect effect of cognitive reappraisal scores, moderated by ELS exposure, on the association between IC11 and wellbeing was significant (a + b), indicating that an increase in the usage of cognitive reappraisal strengthened the association between IC11 and wellbeing, but only for individuals with ELS exposure. **C** The moderated mediation model testing for the conditional indirect effect of expressive suppression scores, moderated by ELS exposure, on the association between IC5 and wellbeing was not significant (a + b). Solid lines = significant associations; dotted lines = nonsignificant associations; *p < 0.05.

In order to perform our moderated mediation analyses, we first checked for significant associations between the five SBM components and the ERQ subscale scores (cognitive reappraisal and expressive suppression). This resulted in significant associations between IC5 and the expressive suppression subscale, and between IC11 and the cognitive reappraisal subscale, which were then taken to moderated mediation modelling of two separate models (see Fig. 3).

Results from the moderated mediation analyses indicated that the relationship between loading coefficients for IC11 and wellbeing was significantly mediated by ERQ reappraisal, depending on ELS exposure; this was not significant for IC5. For IC11, in line with our hypothesis, the reappraisal scores differed depending on ELS exposure, which affected the wellbeing scores (i.e., the conditional indirect effect was significant; path a_3_ + b: *b* = 1.32; SE = 0.627; *p* = 0.035; 95% CI [0.090, 2.55]; see Fig. 3B). More specifically, increases in IC11 loading coefficients was associated with increases in wellbeing through increases in emotion reappraisal, and this effect was only significant for participants exposed to ELS (*b* = 0.902; SE = 0.383; *p* = 0.018; 95% CI [0.152, 1.65]) but not for those not exposed to ELS (*p* = 0.385). There was also a strong association between reappraisal and wellbeing (path b: *b* = 4.01; SE = 0.782; *p* < 0.001; 95% CI [2.48, 5.55]). No other paths were significant (see S1 in Supplemental Materials).

The bootstrapped model examining the relationship between IC5 loading coefficients and wellbeing showed that the index of moderation mediation was not significant (path a_3_ + b: *p* = 0.634), indicating no evidence of a moderating effect of ELS exposure on the hypothesised mediation via expressive suppression strategy (see Fig. 3C). However, the conditional direct effect of ELS exposure on the IC5-wellbeing association was significant (path c_3_: *b* = 4.45; SE = 1.19; *p* < 0.001; 95% CI [2.11, 6.79]), as indicated in the earlier moderation analyses.

## Discussion

Our key findings show that exposure to ELS moderates the relationship between wellbeing and changes in grey matter volume in middle frontal gyri, temporal regions, cingulate cortex, inferior and superior parietal lobules, somatosensory regions, precuneus, and the insula in a relatively large cohort of healthy twins. In addition, in individuals with exposure to ELS, cognitive reappraisal abilities (an antecedent-focused emotion regulation strategy) mediated the relationship between wellbeing and grey matter volume in temporo-parietal regions, precuneus, inferior and middle frontal gyri, inferior and superior temporal gyri, and superior parietal lobule. To the best of our knowledge, this is the first study to report potential multivariate anatomical patterns directly linked to levels of wellbeing in a sample of healthy participants, and further show evidence for a mediating role of emotion regulation on the brain-wellbeing relationship.

Previous studies examining the neural bases of resilience have mainly focused on the relationship between resilience and the brain, without taking ELS exposure into account [19, 35], or focusing on discrete regions of interest [3, 36], or by comparing ELS-exposed versus non-exposed healthy participants, with ‘healthy’ being defined as an absence of psychopathology alone, without taking into account that wellbeing levels range from low to high [4, 16, 37]. Our finding of multivariate patterns of grey matter covariation (GMC) associated with levels of wellbeing and moderated by ELS exposure bridges the gaps between these lines of research, and shows that there may be compensatory mechanisms across multiple structural networks in ELS-exposed participants that lead them to higher wellbeing levels despite their previous exposure. Importantly, this is observed within a group of non-clinical adults, which suggests that morphological changes are associated with wellbeing beyond a mere absence of psychopathology, and highlights a possible neural signature of resilience.

Decreased GMC in the precuneus, posterior cingulate cortex, inferior and superior parietal lobules, somatosensory cortex, and superior temporal gyrus was differentially associated with wellbeing in participants without ELS exposure. The pattern of the association with wellbeing was such that non-exposed participants with higher levels of wellbeing displayed *decreased* GMC in these regions compared to participants with lower levels of wellbeing. This is consistent with functional imaging studies showing reduced connectivity metrics between posterior cingulate cortex and parahippocampal gyrus as a potential biomarker for resilience when comparing clinical groups with healthy participants during a mood induction task [38] and reduced temporal resting-state connectivity at rest [39], suggesting that a decrease in functional connectivity across brain regions may be a protective mechanism in constraining psychopathology and building resilience [40]. Considering the overlap between structural and functional neural networks [41], it may be that decreased coherence, connectivity, or covariation metrics across regions, is a common neural manifestation observed in individuals with high levels of wellbeing.

However, the studies mentioned above do not consider previous exposure to stress/trauma, which is imperative in defining the concept of resilience. Here, by using ELS exposure status as a moderating variable, we observed two covarying networks (IC10 and IC13) that were significantly associated only with ELS-exposed participants. The pattern of association with wellbeing for IC10 indicated that those with high levels of wellbeing (and therefore considered resilient) displayed stronger GMC for a network comprising the precuneus, fusiform gyrus, and the temporal lobe, and a more negative covariation for the inferior and superior parietal lobules. For IC13, resilient participants showed stronger GMC across the somatosensory and motor cortices, as well as the insula and temporal regions, while displaying weaker covariation in the middle frontal and middle temporal gyrus. Stronger covariation across grey matter regions has previously been taken as a proxy for increased structural connectivity, based on the hypothesis that neurons that are connected to each other are likely to have positively correlated volumes [42–44]. Therefore, our finding that resilient individuals exhibit increased GMC in regions broadly associated with emotional and cognitive functioning suggests that resilience may be characterised by stronger connections within higher-level salience and executive control networks. This is in line with the finding that a highly integrated connectome is a primary factor associated with various measures of positive living [45], which lead to higher wellbeing and resilience. In particular, given the insula’s role as an integral hub crucial for a wide range of functions including interoception and self-regulation [46], its coupling with temporal and other somatosensory/motor regions suggests a greater level of awareness of self, other, and the environment. This further ties in with previous studies that show increased insular activity [47] and increased grey matter concentration in the temporal regions [48, 49] in individuals who practice mindfulness meditation, which is an activity that is strongly linked with wellbeing [47, 50].

A psychological factor often implicated in mental health is the ability to regulate emotions, with cognitive reappraisal being linked to adaptive consequences, while expressive suppression has been shown to lead to detrimental outcomes, such as an increase in negative mood symptoms. Although multiple studies examining grey matter volume and cortical thickness have reported associations between emotion regulation strategies and regions such as the prefrontal cortex (PFC), anterior cingulate, insula, and amygdala [51–53], an explicit link with wellbeing is lacking. To this end, our hypothesis was partially supported with a significant mediating effect of cognitive reappraisal on the brain-wellbeing relationship. More specifically, for those exposed to ELS, stronger GMC across the temporo-parietal regions, middle and inferior frontal gyri (in the PFC), and the cerebellum, and weaker GMC across fusiform, postcentral, and inferior temporal gyri, as well as the cerebellum was associated with increases in wellbeing indirectly through increases in cognitive reappraisal scores. This suggests stronger structural covariance across regions involved in cognitive control (such as the dorsolateral and ventromedial PFC), which may be necessary for greater top-down control of activity in the posterior and subcortical regions that are involved in emotion regulatory processes (such as the posterior parietal cortex; Ochsner et al [54]). On the other hand, we speculate that smaller GMC across sensory integration regions (such as fusiform and postcentral gyri) may indicate a down-regulation of information processing via weaker connections, which is adaptive in negative situations [55, 56]. These results are consistent with previous studies employing cognitive reappraisal tasks that show increased neural activity in the PFC and parietal regions for healthy participants [57] and decreased activity in clinical patients [58]. Most importantly, we provide evidence that an adaptive form of emotion regulation mediates the relationship between brain morphology and wellbeing. Interestingly, multiple positive psychology interventions often utilise strategies that include cognitive reappraisal in order to increase one’s wellbeing (e.g. mindfulness) [59–61]. The present results indicate an underlying neural network subserving this emotion-wellbeing behavioural link, and that this network is particularly observable in more resilient individuals, who may either naturally adopt reappraisal strategies that lead to higher wellbeing, or have a high level of wellbeing that allows them to use reappraisal strategies.

There are a number of limitations that need to be considered. First, we dichotomised ELS exposure as either ‘yes’ or ‘no’ to exposure due to the larger number of participants with only 0 (25%) or 1 or 2 events (54%). Future studies may want to consider a continuous approach to the number of ELS events, which may show differential effects on the brain-wellbeing relationship depending on the number of ELS stressors experienced. More specifically, when possible, future studies should explore a ‘loading’ effect in terms of the number of stressors experienced. Furthermore, other stress-related variables such as the age range, frequency, and the duration of ELS events were not taken into consideration, as these require further hypotheses testing and analyses that are beyond the scope of the current study. There is also concern regarding the methodology of using retrospective self-reporting of ELS events; however, dichotomising ELS exposure may have mitigated the potential consequences of under- or over-reporting such events to some extent. Lastly, the linear mixed models utilised in the current study were exploratory and will require replication from an independent sample.

In conclusion, using multivariate SBM analysis we identified differential grey matter volume associated with wellbeing depending on participant’s ELS exposure. Stronger grey matter covariations particularly in the prefrontal and parietal regions in ELS-exposed participants with high levels of wellbeing may indicate possible neural signatures of resilience. Additionally, the application of a cognitive reappraisal strategy could be targeted for neurotherapeutic interventions to promote resilience. Future longitudinal work of ostensibly healthy participants who experienced ELS, especially those with lower levels of wellbeing, may indicate vulnerability rather than resiliency. Lastly, the potential effects of different early life stressor subtypes (e.g., abuse versus health problems) on the brain-wellbeing relationship could further be investigated.

## Supplementary information

Supplementary Materials

## Data Availability

The data and materials used in the current study are available upon reasonable request.
