# Supplementary material for: Grey matter covariation and the role of emotion reappraisal in mental wellbeing and resilience after early life stress exposure
Source: Transl Psychiatry. 2022 Feb 26;12:85. doi: 10.1038/s41398-022-01849-6 (PMC8882193; doi:10.1038/s41398-022-01849-6)
Supplement: Supplementary file 1 — Supplementary Materials [file 41398_2022_1849_MOESM1_ESM.docx]

**Supplementary Materials for**

**Grey matter covariation and the role of emotion reappraisal in mental wellbeing and resilience after early life stress exposure**

Haeme R.P. Park^1,2^, Yann Quidé^1,2,3^, Peter R. Schofield^1,4^, Leanne M. Williams^5^, Justine M. Gatt^1,2^

^1^ Neuroscience Research Australia, Sydney, NSW, Australia

^2^ School of Psychology, University of New South Wales, Sydney, NSW, Australia

^3^ School of Psychiatry, University of New South Wales, Sydney, NSW, Australia

^4^ School of Medical Science, University of New South Wales, Sydney, NSW, Australia

^5^ Psychiatry and Behavioral Sciences, Stanford School of Medicine, Stanford University, Stanford, California, USA

This document contains:

Supplementary Results S1 ‘Loading’ effect analysis; S2 Moderated mediation analyses

Supplementary Results Figures S1 and S2; Table S1

Corresponding author:

Haeme Park

Neuroscience Research Australia

Margarete Ainsworth Building

Barker Street

Randwick 2031

Australia

Email: h.park@neura.edu.au

Phone: +61 2 9399 1884

*S1 ‘Loading’ effect analysis.*

Additional post-hoc analysis was conducted to examine a potential ‘loading’ effect of ELS events, due to the distrbution of the number in the current sample (see Figure S1). We divided the sample into ‘No ELS’ (0 ELS events; n = 61), ‘Low ELS load’ (1-2 events; n = 131), and ‘High ELS load’ (3+ events; n = 50) groups. We then ran linear mixed models and moderated mediation models to examine the effect of ELS group on wellbeing. Similar to the current analysis (e.g., dividing ELS into ‘No ELS’ and ‘Yes ELS’ groups), we found three components that showed a significant ELS group x wellbeing interaction (IC5 *p* < .001; IC10 *p* = .020; IC13 *p* = .037), and the other two components trending towards a significant ELS group x wellbeing interaction (IC2 *p* = .058; IC11 *p* = .083). When we ran our moderated mediation with three levels of ELS moderator (none/low/high), and considered the simple slopes for each ELS group for the brain-wellbeing association mediated by emotion regulation, we observed a significant conditional indirect effect of ELS on wellbeing in the ‘High ELS’ group (*p* = .036), but not in the ‘Low ELS’ (*p* = .055) or ‘no ELS’ (*p* = .772) groups; the overall index of moderation was not significant (*p* = .146). This hints at a ‘loading’ effect but may be affected by reduced statistical power due to the separation of the ELS participants into ‘low’ and ‘high’ groups, and having fewer participants in the ‘High ELS’ group. Such an effect could be revealed in future studies that include a greater proportion of participants with 3+ stressors and a greater exposure to adversity.


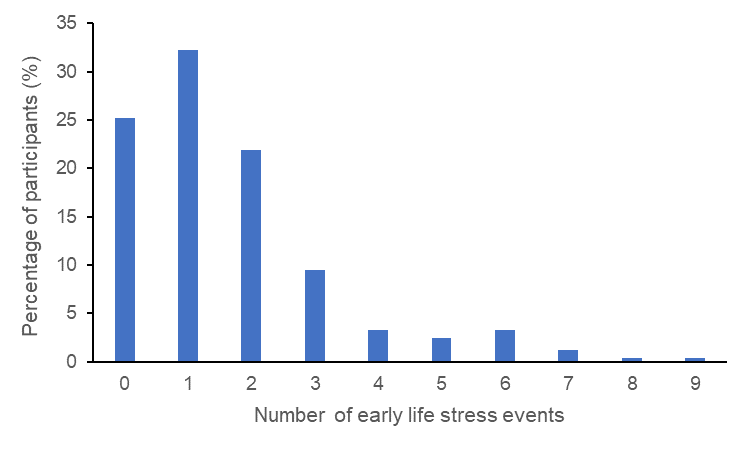


*Figure S1.* A bar graph showing the distribution of the number of early life stress events present in our current sample.

*S2 Moderated mediation analyses.*

**For IC11:** In full, the cognitive reappraisal score was not directly associated with IC11 (a_1_; *p* = .074) or with ELS exposure (path a_2_; *p* = .723). The bootstrapped model also indicated that there was a moderating effect of ELS exposure on the association between IC11 loading coefficients and reappraisal score (path a_3_; *b* = .329; SE = .140; *p* = .019; 95% CI [.055, .603]). Neither the direct effect of IC11 loading coefficients on wellbeing (path c’: *p* = .154) nor the conditional direct effect of ELS exposure on the association between IC11 and wellbeing were significant (path c_3_: *p* = .153). In sum, for IC11, there was an increase in wellbeing scores as the loading coefficients for this component increased, indirectly through increases in the cognitive reappraisal scores *only* for ELS-exposed participants.

**For IC5:** The expressive suppression score was not associated with IC5 (path a_1_: *p* = .270) or with ELS exposure (a_2_: *p* = .787). Additionally, there was no moderating effect of ELS exposure on the association between IC5 and suppression score (path a_3_: *p* = .621). Accounting for IC5, there was a significant association between the suppression and wellbeing scores (path b: *b* = -1.59; SE = .570; *p* = .005; 95% CI [-2.71, -.472]), and a direct effect of IC5 on wellbeing (path c’: *b* = -7.11; SE = 2.17; *p* = .001; 95% CI [-11.4, -2.86]), as well as a conditional direct effect of ELS exposure on the IC5-wellbeing association (i.e., a moderating effect; path c_3_: *b* = 4.45; SE = 1.19; *p* < .001; 95% CI [2.11, 6.79]). There was no mediating effect of suppression scores on the association between IC5 and wellbeing, regardless of ELS exposure (i.e., the indirect effect via suppression score was not significant; path a_1_+b: *p* = .319). In sum, for IC5, there was a direct increase in wellbeing scores as the loading coefficients for this component decreased *only* for participants without ELS exposure, *regardless* of the expressive suppression scores.

| Table S1. Independent components determined by minimum description length criteria. | | | | | |
| --- | --- | --- | --- | --- | --- |
| Component | Voxel directionality | Brain region labels | Broadmann area | L/R volumes (cm^3^) | L/R: max z-value (x,y,z) |
| IC1 | Positive | Thalamus | - | 6.8/7.3 | 12.7 (-10, -16, 10)/11.2 (12, -14, 10) |
|  |  | Inferior Parietal Lobule | 2, 40 | 3.0/3.7 | 4.7 (-39, -33, 42)/8.1 (33, -46, 44) |
|  |  | Extra-Nuclear | - | 2.4/2.1 | 10.0 (-1, -16, 8)/8.4 (3, -11, 10) |
|  |  | Sub-Gyral | 7, 40 | 1.6/2.6 | 4.7 (-40, -33, 43)/8.4 (31, -45, 42) |
|  |  | Lateral Ventricle | - | 1.2/* | 6.4 (-1, -7, 4)/* |
|  |  | Postcentral Gyrus | 1, 2, 5, 40 | 1.1/* | 3.8 (-43, -28, 43)/* |
|  |  |  |  |  |  |
|  | Negative | Fusiform Gyrus | 18, 19, 20, 36, 37 | 1.2/1.1 | 3.7 (-25, -85, -11)/4.1 (42, -31, -17) |
|  |  |  |  |  |  |
| IC2 | Positive | Precuneus | 7, 19, 23, 31, 39 | 9.8/9.3 | 9.3 (-3, -52, 34)/8.8 (1, -53, 39) |
|  |  | Cingulate Gyrus | 23, 24, 31 | 4.0/3.8 | 9.0 (-7, -45, 35)/8.4 (3, -49, 38) |
|  |  | Inferior Parietal Lobule | 7, 39, 40 | 2.9/4.7 | 4.2 (-39, -36, 40)/6.7 (36, -39, 41) |
|  |  | Superior Temporal Gyrus | 13, 22, 39, 41 | 2.3/2.1 | 5.1 (-43, -51, 22)/4.6 (48, -55, 29) |
|  |  | Posterior Cingulate | 23, 29, 30, 31 | 1.4/1.1 | 6.9 (-1, -53, 25)/6.4 (3, -51, 25) |
|  |  | Postcentral Gyrus | 3, 5, 7, 40 | 1.3/1.3 | 4.5 (-6, -48, 63)/4.1 (16, -43, 67) |
|  |  | Superior Parietal Lobule | 7, 40 | 1.2/2.4 | 5.0 (-3, -64, 53)/4.7 (34, -57, 50) |
|  |  | Supramarginal Gyrus | 40 | 1.2/* | 5.0 (-45, -51, 22)/* |
|  |  | Sub-Gyral | 7, 31, 40 | */2.2 | */6.5 (34, -40, 42) |
|  |  |  |  |  |  |
| IC3 | Positive | Declive | - | 6.0/6.6 | 7.3 (-46, -59, -22)/9.6 (48, -56, -22) |
|  |  | Culmen | - | 4.6/6.0 | 6.2 (-42, -52, -23)/9.3 (46, -52, -28) |
|  |  | Tuber | - | 3.8/4.7 | 7.7 (-46, -61, -27)/10.0 (48, -57, -26) |
|  |  | Fusiform Gyrus | 19, 20, 37 | 3.2/3.6 | 6.1 (-46, -61, -18)/7.8 (46, -59, -18) |
|  |  | Middle Temporal Gyrus | 19, 20, 21, 22, 37, 39 | 2.4/1.0 | 4.5 (-46, -59, 14)/4.0 (46, -62, 5) |
|  |  | Cerebellar Tonsil | - | 1.7/3.1 | 7.2 (-46, -60, -31)/9.5 (48, -57, -31) |
|  |  |  |  |  |  |
|  | Negative | Precuneus | 7, 19, 31 | 2.9/2.3 | 4.0 (-13, -71, 34)/4.2 (18, -64, 28) |
|  |  |  |  |  |  |
| IC4 | Positive | Precentral Gyrus | 3, 4, 6, 9 | 4.9/7.4 | 5.7 (-37, -20, 62)/7.3 (39, -25, 62) |
|  |  | Postcentral Gyrus | 1, 2, 3, 5, 7, 40 | 4.5/7.3 | 5.3 (-39, -25, 62)/7.1 (40, -26, 61) |
|  |  | Medial Frontal Gyrus | 6 | 3.0/2.5 | 5.7 (-1, -27, 66)/5.2 (3, -27, 66) |
|  |  | Inferior Parietal Lobule | 40 | 2.9/1.5 | 5.4 (-36, -40, 41)/4.2 (58, -41, 24) |
|  |  | Sub-Gyral | 6, 40 | 1.9/* | 5.4 (-34, -40, 41)/* |
|  |  | Middle Frontal Gyrus | 6, 8 | 1.5/1.5 | 5.1 (-19, -7, 61)/5.5 (21, -6, 61) |
|  |  | Superior Frontal Gyrus | 6 | 1.5/1.3 | 4.9 (-19, -7, 63)/5.3 (21, -7, 63) |
|  |  | Paracentral Lobule | 4, 5, 6 | 1.4/* | 6.0 (-3, -32, 67)/* |
|  |  |  |  |  |  |
|  | Negative | Middle Temporal Gyrus | 20, 21, 37 | 2.6/* | 5.1 (-65, -28, -7)/*s |
|  |  |  |  |  |  |
| IC5 | Positive | Lingual Gyrus | 17, 18, 19 | 5.4/5.3 | 8.2 (-1, -81, 5)/7.9 (1, -81, 5) |
|  |  | Middle Occipital Gyrus | 18, 19, 37 | 5.2/5.1 | 5.3 (-28, -87, 8)/5.2 (34, -80, 11) |
|  |  | Posterior Cingulate | 18, 23, 29, 30, 31 | 2.9/4.1 | 6.7 (-1, -69, 15)/7.7 (6, -69, 15) |
|  |  | Precuneus | 7, 19, 23, 31 | 2.3/3.6 | 7.1 (0, -73, 22)/7.3 (3, -73, 22) |
|  |  | Cuneus | 7, 17, 18, 19, 23, 30 | 14.2/15.5 | 8.4 (-1, -83, 7)/8.5 (10, -74, 13) |
|  |  | Sub-Gyral | - | 1.2/1.6 | 4.9 (-31, -56, 40)/4.8 (37, -75, 12) |
|  |  | Middle Temporal Gyrus | 19, 20, 21, 22, 37, 39 | 1.2/1.1 | 4.4 (-45, -63, 1)/4.7 (40, -74, 13) |
|  |  | Inferior Occipital Gyrus | 17, 18, 19 | 1.2/* | 4.6 (-10, -92, -7)/* |
|  |  |  |  |  |  |
| IC6 | Positive | Middle Frontal Gyrus | 6, 8, 9, 10, 46 | 17.1/13.7 | 10.0 (-36, 24, 38)/11.9 (36, 28, 33) |
|  |  | Superior Frontal Gyrus | 6, 8, 9, 10, 11 | 3.1/3.9 | 8.1 (-33, 34, 30)/9.9 (36, 34, 31) |
|  |  | Precentral Gyrus | 6, 9 | 1.2/* | 9.9 (-36, 24, 36)/* |
|  |  |  |  |  |  |
|  | Negative | Superior Temporal Gyrus | 38, 39 | 1.3/* | 3.0 (-40, 18, -26)/* |
|  |  |  |  |  |  |
| IC7 | Positive | Inferior Semi-Lunar Lobule | - | 5.9/6.5 | 9.3 (-36, -63, -42)/10.1 (37, -63, -41) |
|  |  | Cerebellar Tonsil | - | 5.8/5.7 | 9.6 (-37, -59, -42)/10.1 (37, -62, -42) |
|  |  | Pyramis | - | 2.9/3.5 | 6.7 (-36, -75, -34)/6.4 (39, -72, -34) |
|  |  | Uvula | - | 1.2/2.2 | 4.0 (-16, -76, -34)/5.0 (25, -81, -26) |
|  |  | Tuber | - | 1.0/1.2 | 5.3 (-30, -79, -30)/5.5 (28, -80, -30) |
|  |  |  |  |  |  |
|  | Negative | Posterior Cingulate | 18, 29, 30, 31 | 2.1/1.1 | 7.1 (-22, -59, 8)/5.3 (22, -56, 10) |
|  |  |  |  |  |  |
| IC8 | Positive | Inferior Semi-Lunar Lobule | - | 3.3/3.2 | 14.2 (-1, -57, -41)/14.1 (1, -57, -41) |
|  |  | Cerebellar Tonsil | - | 3.2/3.3 | 13.9 (-1, -56, -41)/13.7 (1, -56, -41) |
|  |  | Uvula | - | 1.3/1.5 | 7.2 (-6, -60, -34)/7.0 (6, -60, -34) |
|  |  | Nodule | - | 1.1/1.2 | 8.9 (-1, -54, -30)/8.8 (0, -54, -30) |
|  |  | Postcentral Gyrus | 2, 3, 4 | 1.0/* | 4.0 (-52, -20, 33)/* |
|  |  |  |  |  |  |
|  | Negative | Culmen | - | 1.7/* | 3.3 (-30, -32, -19)/* |
|  |  | Middle Frontal Gyrus | 6, 8, 9 | 1.0/* | 4.6 (-37, 9, 42)/* |
|  |  |  |  |  |  |
| IC9 | Positive | Anterior Cingulate | 10, 24, 32, 33 | 6.2/7.4 | 8.0 (-1, 38, -1)/8.5 (3, 29, 18) |
|  |  | Cingulate Gyrus | 9, 24, 31, 32 | 4.6/4.5 | 6.2 (-1, 23, 26)/7.0 (3, 22, 27) |
|  |  | Superior Temporal Gyrus | 21, 22, 38 | 4.4/2.0 | 7.1 (-22, 6, -31)/5.3 (22, 6, -31) |
|  |  | Uncus | 20, 28, 34, 36, 38 | 4.2/3.5 | 7.1 (-22, 4, -32)/5.5 (25, -5, -33) |
|  |  | Middle Temporal Gyrus | 20, 21, 22, 38, 39 | 2.0/* | 4.9 (-31, 1, -39)/* |
|  |  | Sub-Gyral | 21 | 1.8/* | 6.9 (-24, 3, -33)/* |
|  |  | Middle Frontal Gyrus | 6, 9, 11, 47 | 1.4/* | 3.6 (-24, 40, -13)/* |
|  |  | Medial Frontal Gyrus | 9, 10, 11, 32 | */1.0 | */6.6 (10, 43, 10) |
|  |  |  |  |  |  |
|  | Negative | Middle Frontal Gyrus | 6, 8, 9 | */1.0 | */3.9 (39, 8, 41) |
|  |  |  |  |  |  |
| IC10 | Positive | Middle Temporal Gyrus | 19, 20, 21, 22, 37, 38, 39 | 13.3/9.9 | 6.4 (-61, -33, -12)/5.3 (67, -24, -6) |
|  |  | Superior Temporal Gyrus | 13, 21, 22, 38, 39, 41, 42 | 9.0/7.9 | 5.3 (-62, -14, 2)/6.6 (62, -37, 18) |
|  |  | Inferior Temporal Gyrus | 19, 20, 21 | 3.7/2.6 | 6.1 (-61, -14, -17)/4.7 (59, -18, -18) |
|  |  | Sub-Gyral | 37 | 1.8/* | 5.0 (-43, -54, -6)/* |
|  |  | Precuneus | 7, 31 | 1.4/* | 4.0 (-16, -62, 22)/* |
|  |  | Fusiform Gyrus | 20, 37 | 1.0/* | 4.8 (-52, -6, -26)/* |
|  |  |  |  |  |  |
|  | Negative | Inferior Parietal Lobule | 7, 39, 40 | 3.7/1.4 | 7.3 (-31, -52, 43)/6.4 (33, -46, 44) |
|  |  | Sub-Gyral | 40 | 2.4/2.7 | 7.6 (-31, -52, 41)/6.8 (33, -46, 41) |
|  |  | Superior Parietal Lobule | 7 | 1.0/* | 6.7 (-28, -50, 43)/* |
|  |  |  |  |  |  |
| IC11 | Positive | Precuneus | 7, 18, 19, 31, 39 | 4.3/2.1 | 8.6 (-24, -64, 38)/8.0 (28, -61, 38) |
|  |  | Sub-Gyral | 9, 21, 37, 39 | 3.7/4.4 | 8.0 (-27, -65, 32)/7.7 (31, -73, 23) |
|  |  | Middle Temporal Gyrus | 19, 21, 22, 37, 39 | 2.1/1.9 | 5.8 (-42, -67, 6)/6.3 (31, -70, 21) |
|  |  | Middle Occipital Gyrus | 19, 37 | 1.5/* | 6.5 (-27, -78, 20)/* |
|  |  | Superior Parietal Lobule | 7 | 1.2/* | 7.5 (-22, -62, 43)/* |
|  |  | Cuneus | 17, 18, 19 | 1.2/* | 6.7 (-25, -79, 22)/* |
|  |  | Superior Temporal Gyrus | 13, 21, 22, 39 | 1.1/1.5 | 4.2 (-45, -50, 19)/5.2 (48, -43, 21) |
|  |  | Cerebellar Tonsil | - | 1.1/1.2 | 3.9 (0, -56, -38)/5.4 (6, -47, -34) |
|  |  | Culmen | - | */2.5 | */6.9 (7, -42, -18) |
|  |  | Declive | - | */1.6 | */4.3 (10, -80, -19) |
|  |  | Inferior Frontal Gyrus | 9, 10, 13, 44, 45, 47 | */1.3 | */4.3 (39, 10, 27) |
|  |  | Middle Frontal Gyrus | 6, 8, 9, 10, 11 | */1.2 | */4.4 (37, 12, 27) |
|  |  | Postcentral Gyrus | 2, 3, 40 | */1.2 | */4.2 (37, -29, 41) |
|  |  | Inferior Parietal Lobule | 39, 40 | */1.0 | */5.5 (46, -44, 24) |
|  |  |  |  |  |  |
|  | Negative | Postcentral Gyrus | 2, 43 | 1.1/* | 3.9 (-56, -18, 20)/* |
|  |  | Inferior Temporal Gyrus | 20, 21 | 1.0/* | 4.3 (-40, -13, -32)/* |
|  |  | Culmen | - | */4.9 | */5.7 (34, -40, -22) |
|  |  | Fusiform Gyrus | 18, 19, 20, 37 | */2.7 | */5.6 (34, -49, -14) |
|  |  | Declive | - | */1.8 | */5.5 (33, -53, -14) |
|  |  |  |  |  |  |
| IC12 | Positive | Declive | - | 12.5/9.7 | 7.4 (-13, -75, -11)/6.7 (15, -70, -10) |
|  |  | Culmen | - | 7.5/7.9 | 7.0 (-13, -67, -9)/6.7 (15, -67, -9) |
|  |  | Lingual Gyrus | 18, 19 | 4.2/2.2 | 7.4 (-13, -73, -10)/6.7 (15, -72, -10) |
|  |  | Fusiform Gyrus | 18, 19, 37 | 2.0/1.0 | 6.7 (-19, -78, -13)/5.6 (21, -68, -10) |
|  |  | Uvula | - | 1.4/* | 4.4 (-31, -80, -23)/* |
|  |  | Precuneus | 7, 19, 31, 39 | */1.7 | */3.7 (27, -59, 40) |
|  |  |  |  |  |  |
|  | Negative | Middle Temporal Gyrus | 19, 21, 22, 37, 39 | 4.1/* | 4.5 (-42, -64, 28)/* |
|  |  | Superior Temporal Gyrus | 21, 22, 39 | */1.2 | */3.9 (50, -36, 6) |
|  |  |  |  |  |  |
| IC13 | Positive | Postcentral Gyrus | 1, 2, 3, 4, 40, 43 | 7.5/6.6 | 8.1 (-55, -17, 30)/6.6 (48, -20, 38) |
|  |  | Superior Temporal Gyrus | 13, 21, 22, 39, 41, 42 | 5.3/2.7 | 6.5 (-43, -30, 14)/6.1 (48, -25, 15) |
|  |  | Precentral Gyrus | 3, 4, 6, 9, 13, 42, 43, 44 | 4.6/3.8 | 7.5 (-53, -19, 34)/6.5 (49, -19, 37) |
|  |  | Sub-Gyral | 43 | 3.8/3.2 | 7.0 (-39, 19, 23)/7.9 (39, 16, 25) |
|  |  | Insula | 13, 40, 41 | 3.4/3.1 | 6.5 (-50, -21, 16)/6.2 (45, -25, 15) |
|  |  | Inferior Parietal Lobule | 2, 40 | 2.7/2.6 | 7.1 (-56, -21, 26)/5.3 (55, -28, 28) |
|  |  | Middle Frontal Gyrus | 6, 8, 9, 10, 46 | 1.7/2.0 | 6.4 (-37, 14, 26)/7.6 (39, 14, 26) |
|  |  | Transverse Temporal Gyrus | 41, 42 | 1.5/1.3 | 6.5 (-52, -26, 12)/6.1 (52, -15, 12) |
|  |  | Middle Temporal Gyrus | 21, 22, 37, 39 | 1.0/1.2 | 4.5 (-56, -47, -5)/5.2 (52, -52, 8) |
|  |  |  |  |  |  |
|  | Negative | Middle Temporal Gyrus | 21, 22 | 1.1/* | 4.6 (-52, -15, -8)/* |
|  |  | Medial Frontal Gyrus | 6, 8, 9, 10 | */1.7 | */3.3 (4, 52, 13) |
|  |  | Middle Frontal Gyrus | 8, 9 | */1.9 | */6.2 (36, 28, 33) |
|  |  |  |  |  |  |
| IC14 | Positive | Insula | 13, 40, 41, 45, 47 | 8.6/8.4 | 5.2 (-37, 6, -3)/5.8 (40, -27, 18) |
|  |  | Inferior Frontal Gyrus | 11, 13, 45, 47 | 4.2/6.2 | 5.0 (-34, 25, 0)/5.5 (34, 25, 0) |
|  |  | Extra-Nuclear | 13, 47 | 3.6/2.1 | 4.9 (-34, 23, -1)/5.4 (34, 23, -1) |
|  |  | Parahippocampal Gyrus | 19, 28, 30, 34, 35, 36, 37 | 3.0/* | 3.6 (-22, -14, -13)/* |
|  |  | Superior Temporal Gyrus | 13, 22, 38, 41 | 2.1/1.5 | 5.1 (-40, -30, 17)/4.9 (42, -30, 17) |
|  |  | Medial Frontal Gyrus | 11, 25 | 1.4/1.2 | 5.2 (-12, 12, -16)/5.4 (13, 12, -16) |
|  |  | Subcallosal Gyrus | 13, 25, 34, 47 | 1.1/1.1 | 4.9 (-12, 12, -14)/5.2 (13, 12, -14) |
|  |  | Culmen | - | */1.1 | */3.7 (12, -40, -13) |
|  |  |  |  |  |  |
|  | Negative | Cuneus | 7, 17, 18, 19, 23, 30 | 1.7/2.9 | 4.7 (0, -76, 20)/5.1 (3, -76, 16) |
|  |  | Precentral Gyrus | 4, 6, 9 | */1.2 | */3.5 (61, -2, 22) |
|  |  |  |  |  |  |
| IC15 | Positive | Lentiform Nucleus | - | 5.0/3.7 | 5.0 (-22, 9, -2)/4.8 (25, 6, 2) |
|  |  | Extra-Nuclear | 13 | 3.6/2.7 | 4.8 (-16, 17, -1)/4.5 (16, 19, -1) |
|  |  | Middle Temporal Gyrus | 19, 20, 21, 22, 37, 39 | 3.3/8.9 | 7.3 (-43, -65, 7)/8.9 (45, -62, 9) |
|  |  | Inferior Temporal Gyrus | 19, 20, 37 | 1.6/1.9 | 6.0 (-45, -64, 2)/6.8 (43, -63, 2) |
|  |  | Middle Occipital Gyrus | 19, 37 | 1.6/1.2 | 7.0 (-43, -65, 5)/7.6 (43, -64, 5) |
|  |  | Sub-Gyral | - | 1.3/2.3 | 6.2 (-40, -67, 10)/7.4 (42, -62, 5) |
|  |  | Caudate | - | 1.1/* | 4.8 (-16, 18, 1)/* |
|  |  | Superior Temporal Gyrus | 22, 39, 42 | */2.0 | */5.8 (49, -58, 14) |
|  |  | Precuneus | 7, 19, 39 | */1.0 | */4.1 (37, -74, 34) |
|  |  |  |  |  |  |
|  | Negative | Inferior Frontal Gyrus | 10, 13, 44, 45, 46, 47 | 6.3/7.0 | 5.5 (-42, 26, -4)/5.8 (48, 22, 3) |
|  |  | Posterior Cingulate | 18, 23, 29, 30, 31 | 3.4/3.3 | 8.8 (-22, -58, 8)/7.0 (22, -56, 11) |
|  |  | Sub-Gyral | 10 | 1.6/1.8 | 6.1 (-21, -59, 14)/6.3 (24, -56, 14) |
|  |  | Precuneus | 7, 23, 31 | 1.2/* | 3.7 (-15, -63, 25)/* |
|  |  | Extra-Nuclear | 47 | 1.0/1.2 | 8.6 (-24, -58, 8)/8.1 (25, -55, 10) |
|  |  |  |  |  |  |
| IC16 | Positive | Medial Frontal Gyrus | 6, 8, 9, 10, 11, 32 | 5.1/7.5 | 5.3 (-1, 51, 25)/6.5 (4, 52, 14) |
|  |  | Superior Frontal Gyrus | 6, 8, 9, 10, 11 | 3.6/6.1 | 5.4 (-22, 17, 43)/6.5 (25, 11, 48) |
|  |  | Middle Frontal Gyrus | 6, 8, 9, 10, 46 | 3.0/3.9 | 5.9 (-45, 26, 18)/6.5 (27, 11, 46) |
|  |  | Sub-Gyral | 6, 8, 20 | 2.9/2.6 | 6.4 (-42, 26, 18)/6.7 (25, 13, 46) |
|  |  | Inferior Frontal Gyrus | 9, 10, 44, 45, 46 | 2.0/1.4 | 5.8 (-45, 27, 17)/4.4 (43, 34, 13) |
|  |  |  |  |  |  |
|  | Negative | Cuneus | 17, 18 | 2.1/* | 4.4 (-9, -96, -1)/* |
|  |  | Lingual Gyrus | 17, 18, 19 | 1.7/* | 4.4 (-9, -96, -3)/* |
|  |  | Parahippocampal Gyrus | 19, 35, 36, 37 | 1.2/2.4 | 3.3 (-19, -56, -6)/4.3 (31, -40, -6) |
|  |  | Culmen | - | 1.0/* | 3.3 (-19, -56, -7)/* |
|  |  | Postcentral Gyrus | 1, 2, 3, 4, 5, 43 | */1.7 | */4.3 (56, -16, 27) |
|  |  | Anterior Cingulate | 24, 32 | */1.6 | */4.0 (6, 34, 9) |
|  |  |  |  |  |  |
| Note: L/R: left/right; *: non-significant voxels or voxels with *z<*2.5 and volume smaller than 1cm^3^ | | | | | |


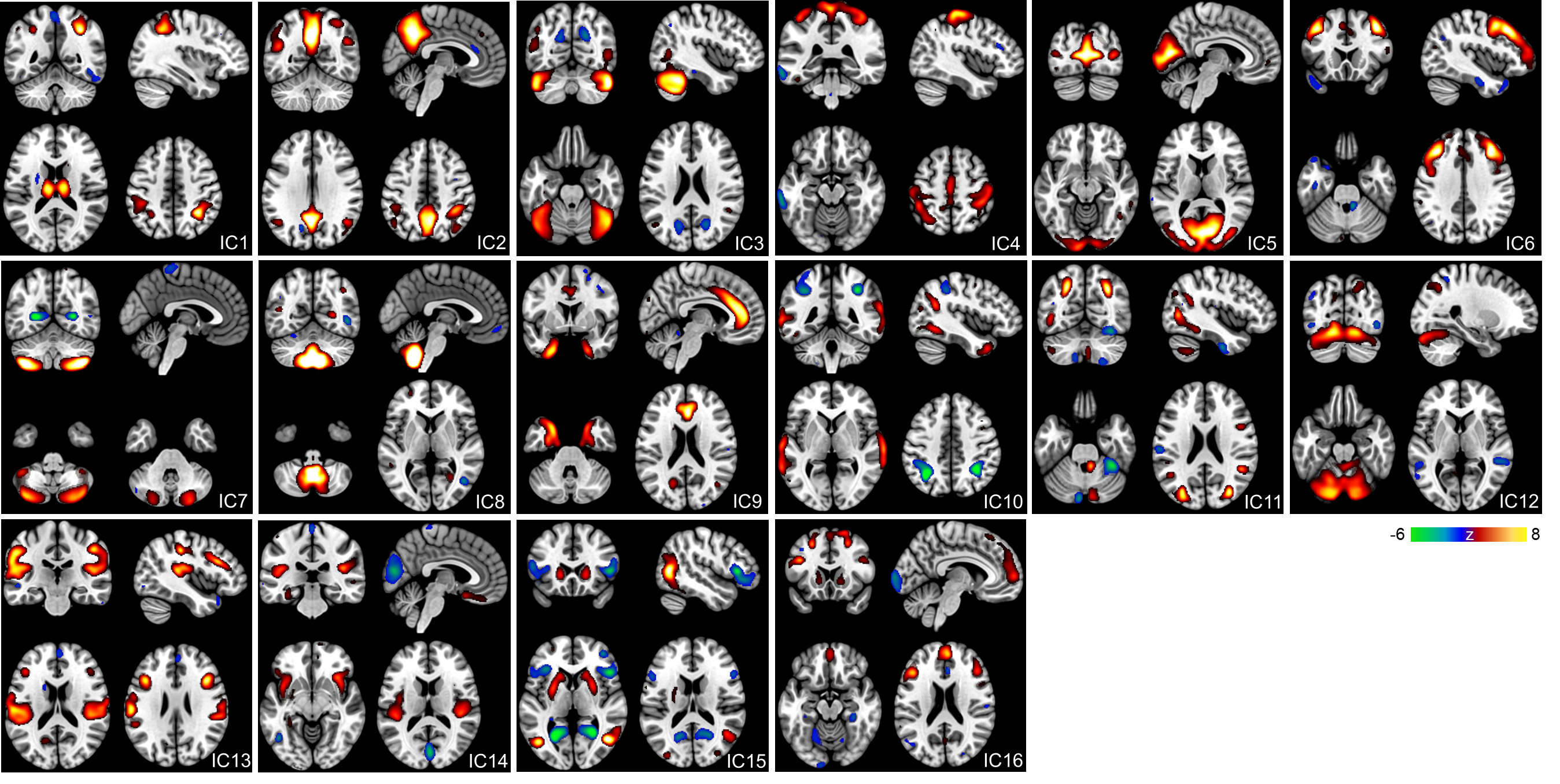


*Figure S2.* Spatial maps of the 16 independent components (IC) derived from source-based morphometry analysis. Warm colours represent positive loading coefficients for the component, while cool colours indicate negative loading coefficients. The components were thresholded at |z| > 2.5; the colour bar indicates the z-value. Images are in neurological convention (left side of the image corresponds to the left hemisphere).
